# Influence of Gas Annealing on Sensitivity of AlN/4H-SiC-Based Temperature Sensors

**DOI:** 10.3390/ma14030683

**Published:** 2021-02-02

**Authors:** Seung-Woo Jung, Myeong-Cheol Shin, Michael A. Schweitz, Jong-Min Oh, Sang-Mo Koo

**Affiliations:** 1 Department Electronic Materials Engineering, Kwangwoon University, 20 Kwangwoon-ro, Nowon-gu, Seoul 01897, Korea; swjung0819@kw.ac.kr (S.-W.J.); smc0753@naver.com (M.-C.S.); michael.schweitz@schweitzlee.com (M.A.S.); jmOh@kw.ac.kr (J.-M.O.); 2 Department of Electrical and Mechanical Engineering, Nagoya Institute of Technology, Gokisocho, Showa Ward, Nagoya, Aichi 466-8555, Japan

**Keywords:** AlN, 4H-SiC, temperature sensor, Schottky barrier diodes, XPS

## Abstract

In this study, the physical and electrical characteristics of an AlN/4H-SiC Schottky barrier diode-based temperature sensor annealed in various gas atmospheres were investigated. An aluminum nitride (AlN) thin film was deposited on a 4H-SiC substrate via radio-frequency sputtering followed by annealing in N_2_ or O_2_ gas. The chemical composition of the film was determined by X-ray photoelectron spectroscopy (XPS) before and after annealing, and its electrical properties were evaluated by plotting a current–voltage (I–V) curve. The voltage–temperature (V–T) characteristics of the sensor were extracted from the current–voltage–temperature (I–V–T) plots constructed in the temperature range between 475 and 300 K in steps of 25 K. Sensitivities of 9.77, 9.37, and 2.16 mV/K were obtained for the as-grown, N_2_-annealed, and O_2_-annealed samples, respectively.

## 1. Introduction

Silicon carbide (SiC) is a promising next-generation semiconductor composed of silicon and carbon. It possesses a wide bandgap (3.2 eV), good physical properties, low leakage current, high thermal conductivity, excellent electron mobility, and high critical electric field [1]. Thus, SiC can be used in high-temperature and high-power electronic devices as well as in inverters of electric vehicles and aerospace applications. In addition, SiC exhibits a high thermal and chemical stability and a small crystal lattice mismatch with aluminum nitride (AlN), which is another wide bandgap semiconductor of high interest. These properties make SiC a most suitable substrate material for epitaxially grown AlN films. AlN is a III–V compound semiconductor with a large bandgap of 6.2 eV, high melting point of 3200 K, high thermal conductivity of 3.2 W/mK, and high thermal and chemical stability, similar to those of SiC [2,3]. The wide bandgap of AlN makes it suitable for photoelectric applications, including deep ultraviolet detectors and various sensors. AlN thin films can be grown by different methods including molecular beam epitaxy, metal organic chemical vapor deposition, pulsed laser deposition, and radio-frequency (RF) sputtering [3]. Sputter deposition enables the growth of large area, highly uniform, inexpensive, and high-quality AlN thin films at relatively low temperatures in a wide deposition range [4,5]. Meanwhile, gas annealing is an important process performed during the fabrication of compound semiconductors, which can increase the AlN crystallinity [6] and reduce the number of defects. The manufactured SBDs show promise for use in combined sensors for measuring both temperature and UV-light. Further sensing capabilities are being explored in related work [7,8,9,10,11,12]. In this study, Schottky barrier diode (SBD)-based temperature sensor samples were fabricated by RF sputtering deposition of AlN onto an n-type 4H-SiC substrate. A number of the samples were subsequently annealed in either a nitrogen or oxygen atmosphere after which sensor parameters were investigated to establish the effects of the applied annealing gas.

## 2. Materials and Methods

**Figure 1:**
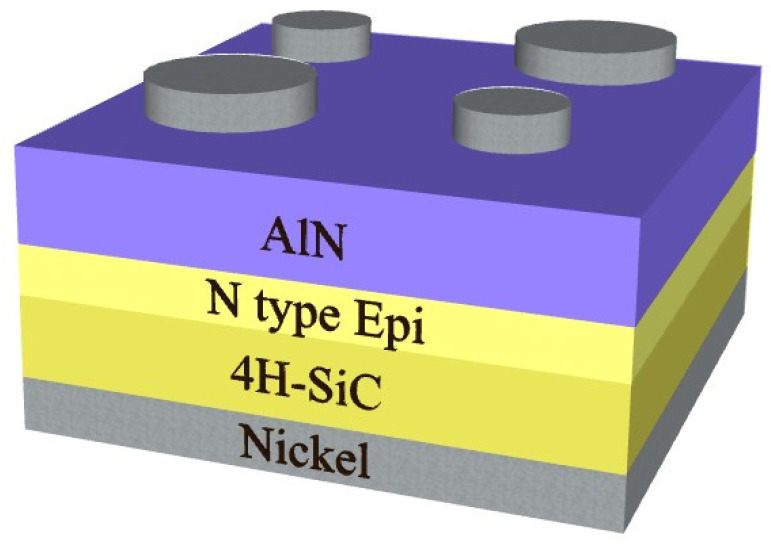
Structure of the fabricated AlN/4H-SiC Schottky barrier diode samples.

AlN thin films were deposited on n-type 4H-SiC substrates containing n-type epitaxial 4H-SiC layers by RF sputtering of an AlN target (99.9%) in an argon atmosphere (99.999%) at room temperature. The sputtering power was 160 W, with a gas flow rate of 4.0 sccm, as maintained by a mass flow controller. The chamber pressure was held at 25 mTorr during the 5 h deposition process. AlN film thickness was measured using a stylus profiler (SP) and atomic force microscopy (AFM). According to [13], AFM thickness measurements are in good agreement with measurements performed by ellipsometry. The resulting 400 nm thick AlN film samples were annealed at 500 °C for 1 h in two different atmospheres (N_2_ or O_2_) using an annealing furnace. Finally, top electrodes of nickel were deposited on the AlN films to a thickness of 60 nm by means of an e-beam evaporator. Then, the samples were subjected to rapid thermal annealing at 950 °C for 60 s in an N_2_ atmosphere to form ohmic contacts. Figure 1 shows the SBD structure of the manufactured devices. The devices fabricated without annealing or with annealing in either a N_2_ or O_2_ atmosphere are herein named “as-grown”, “N_2_-annealed”, and “O_2_-annealed”, respectively. Note that the current level degradation for all the devices remains within less than 10% of the initial level after a six-hour long exposure to elevated temperature (675 K). The detailed results of the comparative stress test study will be discussed elsewhere. The compositions of their AlN films were determined by X-ray photoelectron spectroscopy (XPS) before deposition of the top electrodes. Finally, to evaluate the effects of the different annealing conditions on the temperature sensing properties of the completed devices, their I-V-T characteristics were measured using a Keithley 4200-SCS (Tektronix, Beaverton, OR 97077, U.S.A.) for temperatures ranging from 300 to 475 K.

## 3. Results and Discussion

**Figure 2:**
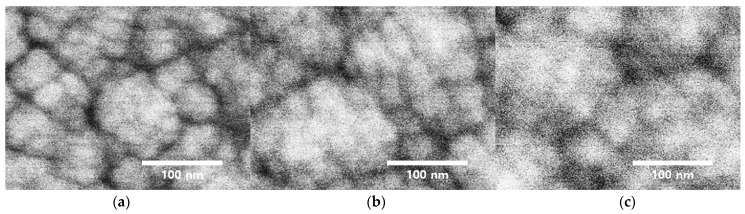
AlN film morphology SEM imaging (**a**) as-grown, (**b**) N_2_-annealed, and (**c**) O_2_-annealed samples.

Figure 2a–c show scanning electron microscopy (SEM) images detailing the surface morphology of the manufactured AlN films. The AlN thin films in Figure 2b,c display larger average grain size relative to the grain size of the film in Figure 2a.

**Figure 3:**
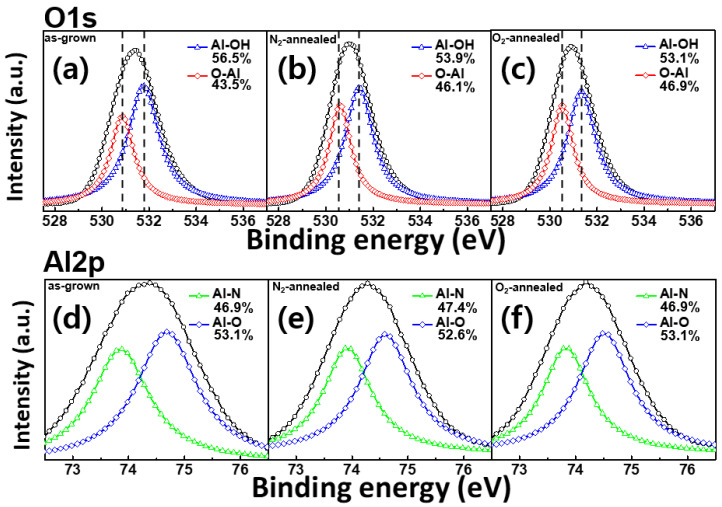
The results of XPS analysis of the fabricated AlN films performed before and after annealing; (**a**–**c**) the O 1s peaks of the various AlN films where (**a**) relates to the as-grown sample, (**b**) the N_2_-annealed sample, and (**c**) the O_2_-annealed sample; (**d**–**f**) the Al 2p peaks with Al–N and Al–O sub-peaks of the different samples, with results in (**d**) for the as-grown sample (**e**), the N_2_-annealed sample, and (**f**) the O_2_-annealed sample.

Figure 3 shows the O 1s and Al 2p XPS spectra of the AlN films obtained for the as-grown sample, as well as samples after annealing in either N_2_ or O_2_ gas. The Al–OH and O–Al peaks in the O 1s spectrum of the as-grown sample are centered at 531.78 and 530.88 eV, respectively. After gas annealing, in the N_2_-annealed sample, Al–OH and O–Al binding energies shifted to 531.38 eV and 530.56 eV, respectively. Said energies were 0.4 eV and 0.32 eV lower than those of the as-grown sample, respectively. Furthermore, in the O_2_-annealed sample, the Al–OH and O–Al binding energies shifted to 531.33 eV and 530.52 eV, which in turn were lower compared with the binding energies of the N_2_-annealed sample. Additionally, the relative area of the Al–OH peak decreased. The Al–N peak of the Al 2p spectrum was detected at 74.68 eV, and the corresponding Al–O peak was centered at 73.78 eV. The ratios between these two components determined for the as-grown and O_2_-annealed samples were identical, while the relative area of the Al–N peak obtained for the N_2_-annealed sample increased to 47.4%. The reason for the higher O 1s binding energy in the as-grown sample than in the annealed samples was likely to be the result of chemisorption of oxygen or superficially adsorbed OH species [14]. The observed decrease in the relative Al–OH peak area after annealing can be attributed to the cleavage of O-H bonds at high temperatures [15]. Similarly, the decreased Al–O peak area in the Al 2p spectrum of the N_2_-annealed sample indicated that N_2_ gas suppressed the formation of Al–O bonds and increased the fraction of Al–N bonds during annealing [14].

**Figure 4:**
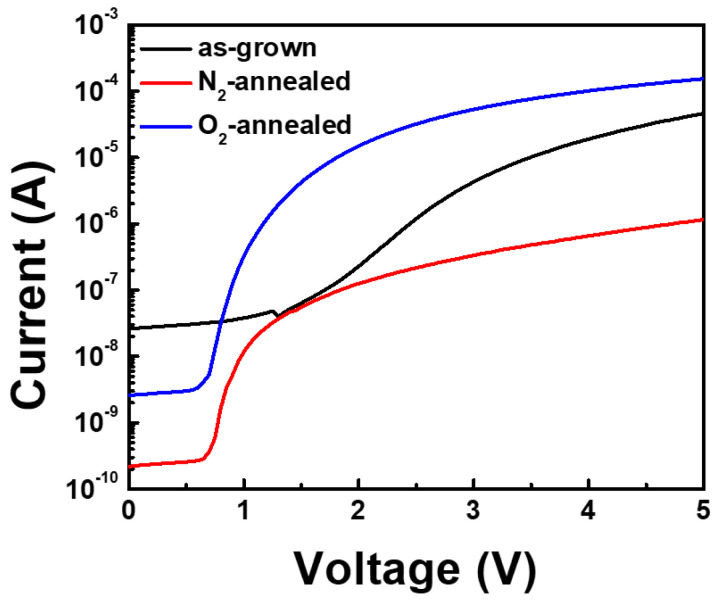
Current–voltage (I-V) characteristics of the AlN film Schottky barrier diode (SBD) devices obtained before and after annealing.

The I–V characteristics of the annealed and non-annealed SBD devices obtained at room temperature are shown in Figure 4. The as-grown sample shows a current of 4.6 × 10^−5^ A at a voltage of 5 V. The corresponding current increased to 1.5 × 10^−4^ A in the N_2_-annealed sample, while it decreased to 1.15 × 10^−6^ A in the O_2_-annealed sample. The XPS analysis indicated that the O 1s binding energy was higher in the as-grown sample than in the annealed samples. As previously mentioned, this is likely to have resulted from chemisorbed surface oxygen or superficially adsorbed OH species [15]. Consequently, the improved electrical characteristics of the N_2_ and O_2_-annealed samples were attributed to the decreased charge carrier trap concentration. Meanwhile, the Al–N peak area in the Al 2p spectrum increased after N_2_ annealing because of the suppression of the Al–O bond formation process. Thus, the purity of the N_2_-annealed sample appears to have increased as a result of the N_2_ gas exposure during annealing [16]. At the same time, it increased the electrical resistance of the AlN layer, thereby reducing its conductivity [17].

**Figure 5:**
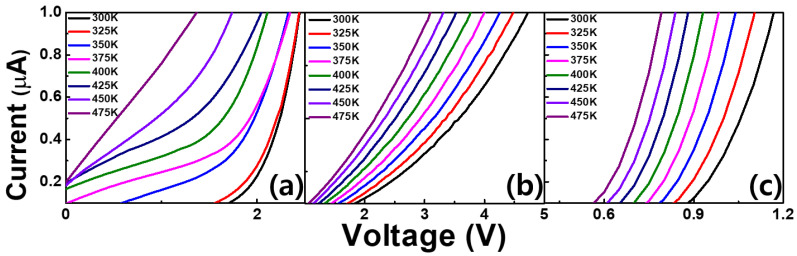
Forward current–voltage–temperature (I-V-T) characteristics of the fabricated AlN/4H-SiC SBD devices: (**a**) as-grown, (**b**) N_2_-annealed, (**c**) O_2_-annealed.

The I-V-T graphs obtained for the three studied samples in the temperature range from 475 to 300 K in steps of 25 K are shown in Figure 5. In these graphs, the turn-on voltage decreases with increasing temperature. Thermionic emission theory describes charge carrier activation at low currents and high temperatures [18]. Its governing equation shows the temperature dependence of the forward voltage:

(1) $$V=IR+\frac{kT\eta }{q}ln\left(\frac{I}{AR^{**}T^{2}}\right)$$

where *η* is the ideality factor, *q* is the electronic charge, *k* is Boltzmann’s constant, *A* is the contact area, *T* is the temperature, and R** is Richardson’s constant. Obviously, the equation was shown to need low voltage in high temperature (Figure 5). The ideality factors for the as-grown, N_2_-annealed, and O_2_-annealed samples are 11.03, 2.79, and 4.65, respectively at 475K. The Richardson constant for AlN is ≈57.6 A cm^−2^ K^−2^ [4]. *R_s_* was calculated from the I-V-T curve. The obtained values were as follows; as-grown sample: 0.0044 Ω; N_2_-annealed sample: 0.331 Ω; O_2_-annealed sample: 0.0187 Ω for 1.75, 1.85, 0.9 V turn-on voltages at 300 K, respectively. Thus, *R_s_* in the as-grown sample was lower than in the annealed sample at 300 K. On the other hand, at 475 K, the resistance of the as-grown sample was 0.015 Ω, while it was 0.2981 Ω in the N_2_-annealed sample and 0.0005 Ω in the O_2_-annealed sample. Consequently, the O_2_-annealed sample exhibited larger currents at elevated temperatures than the other samples. High-performance temperature sensors consume little power and exhibit good linearity and sensitivity [19]. The sensitivities of the fabricated SBD devices were measured at various forward currents.

**Figure 6:**
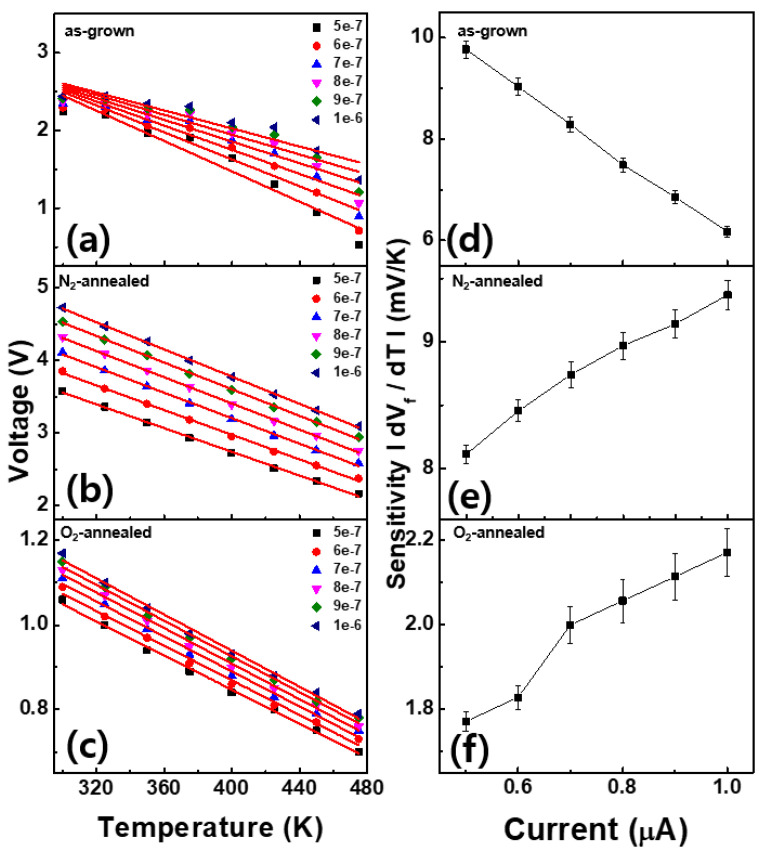
Forward voltage vs. temperature plots for the fabricated AlN/4H-SiC SBD devices at various currents: (**a**) as-grown, (**b**) N_2_-annealed, and (**c**) O_2_-annealed sample. sensitivity vs. current obtained from V-T plots, including error bars; (**d**) as-grown, (**e**) N_2_-annealed, and (**f**) O_2_-annealed samples.

Figure 6 shows the V-T graphs extracted from the respective I-V-T plots at temperatures between 475 and 300 K and currents between 0.5 and 1 A. In all the V-T graphs, the temperature increases with decreasing voltage. For the as-grown sample, the peak current begins to decrease after achieving a maximum sensitivity of 9.77 mV/K at 0.5 µA. In contrast, the N_2_-annealed and O_2_-annealed samples exhibit relatively small sensitivities at low currents; however, their magnitudes increase to 9.37 and 2.16 mV/K at 1 µA, respectively. The sensitivity of the sample fabricated by Ao et al. was approximately 1.3 mV/K [20], which was 4.7 times lower than the value obtained for the as-grown sample in the present study. Furthermore, the N_2_-annealed and O_2_-annealed samples demonstrated the same trends as that observed in the temperature sensor fabricated by Tan et al. The latter possessed a sensitivity of approximately 1.59 mV/K, which was 5.9 and 1.3 times lower than the maximum sensitivities of the N_2_-annealed and O_2_-annealed devices, respectively [21].

## 4. Conclusions

In this study, an AlN/SBD temperature sensor was fabricated by depositing an AlN thin film on an n-type 4H-SiC substrate by RF sputtering. The effects of N_2_ and O_2_ annealing on the composition of the AlN layer and the thermal sensitivity and electrical characteristics of the produced device were examined. The high-temperature annealing process caused the cleavage of Al–OH bonds. As a result, the relative area of the Al–O peak increased, which was apparently because of a decrease in the number of oxygen vacancies. Additionally, N_2_ annealing increased the purity of the AlN film due to the lower number of Al–O bonds, essentially reducing the effective doping of the film. Consequently, the current decreased with the increasing electric resistance that resulted from the N_2_ annealing. Finally, the thermal sensitivity of the fabricated sensor was calculated from its V-T characteristics, which was extracted from the corresponding I-V-T plot. The sensitivity values obtained for the as-grown, N_2_-annealed, and O_2_-annealed samples exceeded the sensitivities of the sensors reported in the literature by 4.7, 5.9, and 1.3 times, respectively.

## Data Availability

Data is contained within the article.
